# Dietary Diversity of an Adult Solomon Islands Population

**DOI:** 10.3390/nu11071622

**Published:** 2019-07-17

**Authors:** Bridget Horsey, Libby Swanepoel, Steven Underhill, Judith Aliakbari, Sarah Burkhart

**Affiliations:** 1 School of Health and Sport Sciences ML41, University of the Sunshine Coast, Locked Bag 4, Maroochydore DC 4558, Queensland, Australia; 2 Australian Centre for Pacific Islands Research, University of the Sunshine Coast, Locked bag 4, Maroochydore DC 4558, Queensland, Australia; 3 School of Natural Resources and Applied Sciences, Solomon Islands National University, Honiara, Solomon Islands; 4 The Hilltop Training Institute, Auki, Malaita, Solomon Islands

**Keywords:** Pacific Islands, malnutrition, food systems, food consumption, food preference, dietary intake

## Abstract

Ongoing dietary transitions in the Solomon Islands has resulted in an over-reliance on commercially sourced foods, leading to food insecurity, and a subsequent rise in multiple forms of malnutrition. The aim of this study was to investigate the individual dietary diversity and food preferences of the adult population living in Auki, Solomon Islands. A cross-sectional study involving 133 adults was undertaken in the Auki district via an interviewer-administered questionnaire. Individual dietary diversity scores (DDS) were determined based on the results of a 24-h recall method. Overall mean DDS was 7.27 (range 2–12). Females and participants who lived outside the Auki town center had significantly higher dietary diversity scores. Low consumption of a variety of nutritious foods within food groups and high consumption of energy dense processed foods, indicates that diet quality is likely limited in some of this population. Participants desire for a diverse diet including local foods suggests that current dietary diversity status in this population may be influenced by food security rather than food preference.

## 1. Introduction

The Pacific Islands are experiencing some of the highest rates of malnutrition in the world [1]. Directly caused by an inadequate dietary intake of energy and/or nutrients, malnutrition is experienced in three forms; undernutrition (including stunting and underweight), overnutrition (including overweight and obesity) and micronutrient deficiencies [1,2,3]. Traditionally, malnutrition in the Pacific Islands has been predominantly characterised by undernutrition and micronutrient deficiencies [1]. However, accelerated changes in dietary patterns, known as nutrition transitions and a lack of high-quality diet is contributing to increasing rates of overnutrition [1]. This has resulted in the coexistence of all forms of malnutrition across the Pacific Islands, commonly referred to as the triple burden of malnutrition [3,4,5,6]. The adverse health outcomes of overnutrition in the Pacific Islands are evidenced by the prevalence of diet related non-communicable diseases such as type 2 diabetes and coronary heart disease [1]. The increasing complexity of this health crisis has severe consequences for national and individual development including high societal and personal costs, and increased morbidity and mortality rates [6,7,8]. In countries like the Solomon Islands where the health system already lacks resources and functions poorly, the triple burden of malnutrition and its consequences creates significant health challenges [6,7,8].

Located to the northeast of Australia and surrounded by the South-West Pacific Ocean, the Solomon Islands archipelago is one of the least developed countries in the Indo-Pacific region [9]. Most of the population of the Solomon Islands reside in rural areas (80%) and are dependent on semi-subsistence-based farming and food sourced from fishing [7]. In contrast, the urban-based population is concentrated in the capital city Honiara or other urban towns such as Auki, and relies on commercial food supply chains [7,10,11]. Dietary patterns in the Solomon Islands have progressively shifted from traditional diets consisting of a variety of fresh fish, tubers and local vegetables, towards a less nutritious, and less varied diet [7]. These dietary changes are driven by limited land access within urban populations, low household incomes and the availability of cheaper, imported and processed foods (for example white rice, canned fish, canned meats and white flour) [7,9,10,11,12]. Furthermore, this ongoing dietary transition in the Solomon Islands, alongside a changing food system, has resulted in an over-reliance on commercially sourced foods, leading to the consumption of a poor variety of foods and a subsequent rise in food and nutrition insecurity [11]. This can result in sub-optimal dietary diversity due to insufficient access to a variety of nutritious foods [12,13]. The consequences of this change in dietary behaviour is reflected in increased rates of maternal and child undernutrition issues alongside elevated levels of overweightness and obesity in the general population [14,15,16]. 

Dietary diversity, described as the range of different foods or food groups consumed over a given period of time, is strongly associated with diet quality [12]. Increasing the variety of nutritious foods eaten promotes optimal health and avoids malnutrition [12,13,16], and diverse diets have been shown to have numerous protective factors against various chronic diseases and morbidity [17,18]. The literature shows that issues of malnutrition in many low-to-middle-income countries, such as the Solomon Islands, are a result of monotonous diets that subsequently increase the risk for malnutrition [12,19]. Also, there is evidence that the Solomon Islands experience high rates of malnutrition [14]. However, there have been few studies investigating consumption [20], and in particular, there is little evidence of measurement of dietary diversity in food systems and the consequent health implications throughout the Pacific region. Previously published literature has been based on the Honiara population [10] or focused on food security implications from food production and market sales [11], aqua-culture [21] and logging [22]. Research investigating consumption (specifically, dietary diversity) would provide insight into the current state of a remote Solomon Island population. Therefore, the aim of this study was to investigate the dietary diversity and food preferences of the adult population living in Auki, Solomon Islands. 

## 2. Materials and Methods 

### 2.1. Location

**Figure 1:**
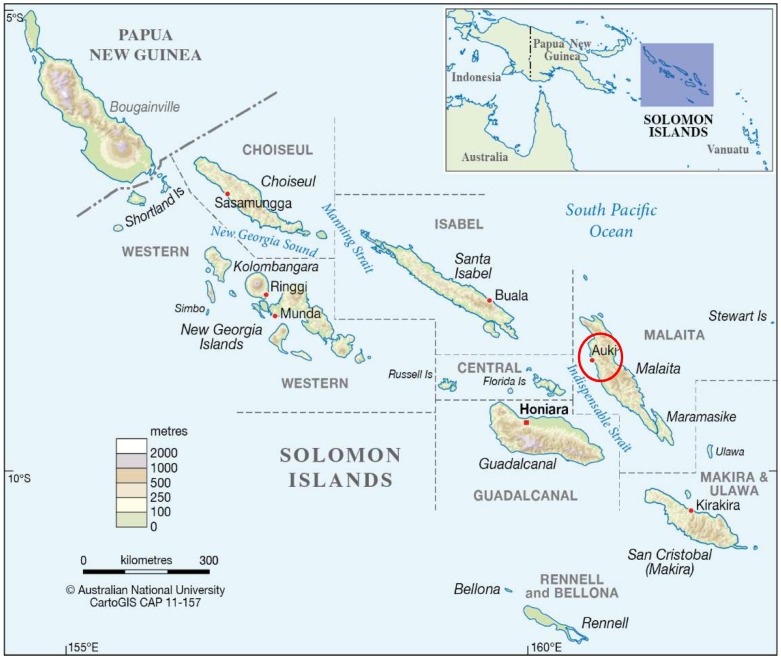
A colour relief map of the main Solomon Islands showing province borders, highlighting Auki’s location and including a location inset (Source: CartoGIS Services, College of Asia and the Pacific, The Australian National University) [26].

This study was undertaken in Auki, the provincial capital of the island of Malaita and one of the largest towns in the country (Figure 1) [23]. According to the national census report in 2009, Auki has a total population of 5105 people, with more females than males and 3098 individuals aged 15 years or older with 210 over the age of 60 [24]. Auki is the major trade link between Honiara and Malaita and is the main source of services and food supplies for rural villages on the island [23]. As a semi-remote and regional center experiencing a high rate of rural-urban migration, Auki’s food systems are likely in active transition from a semi-subsistence to more commercial-focused food supply system [23]. Malaita Island is one of the most socio-economically disadvantaged areas in the Solomon Islands [24], such that economically-driven food choice decisions might affect diet quality regardless of food preferences. Auki’s comparatively small geographic area and population size allowed for an investigation of food consumption and dietary diversity to be undertaken. The lack of any tangible tourism industry in Malaita [25] also removed the risk of any conflicting food systems within the region, with the food production and supply systems almost solely orientated towards the indigenous population [7,22]. Figure 1 shows the location of Auki in the Solomon Islands in relation to Australia.

### 2.2. Recruitment of Participants

This observational cross-sectional study was undertaken between November and December 2018. Participants were voluntarily recruited by a team of five Australian researchers and five local translators. Eligibility criteria included adult residents (aged 18 to 72 years) living in Auki, which included the Auki town center and any surrounding villages within the Auki district. Based on a convenience approach to sampling, participants were approached in markets, homes, shops, and on street side walks in the Auki town center and two of the surrounding villages. The two villages (Lilisiana and Kilusakwalo) were randomly selected for recruitment to ensure that semi-rural residents were represented in the study. A research participant information sheet was available for all participants and the study had ethical approval from the researchers’ institution (approval number: S181248). The interviewer-administered questionnaire was conducted at the place of invitation. 

### 2.3. Data Collection Tool

A semi-structured, interviewer-administered questionnaire divided in three sections; socio-demographic characteristics (section one), dietary diversity (section two) and food preferences (section three) was used to collect data from study participants. After gaining verbal consent to participate, interviewers, each with the assistance of a local translator (native speaker) asked 11 questions from section one, six questions from section two and four questions from section three. 

Dietary diversity (section two) was measured with the Food and Agriculture Organization of the United Nations (FAO) validated Dietary Diversity Questionnaire (DDQ) tool that was adapted to the local context (by including local foods and local language related to meal times) by the lead author and a research team member based in the location [27]. Instructions for administering the DDQ was followed as outlined in the FAO tool [27]. Participants were asked to recall all food and drinks consumed individually over the previous 24-h period, including foods consumed outside of the home. If this period did not depict “usual” intake due to special occasions, feasting or illness, participants were asked to recall a different 24-h period that reflected their typical food consumption (generally the previous days were used). To ensure that complete and accurate food recall was attained, interviewers probed for any food groups, including questions about snacks or added ingredients (for example sugar and salt) that participants may have forgotten. Also, participants were asked to list separate ingredients from composite dishes [27]. The food groups used were:

1. Cereals
2. White tubers and roots
3. Vegetables
4. Fruit
5. Meat
6. Eggs
7. Fish and other seafood products
8. Legumes, nuts and seeds
9. Milk and milk products
10. Oils and fats
11. Discretionary (e.g., foods containing added sugars or highly processed foods with minimal nutritional benefits)
12. Spices, condiments and beverages

The final four questions (section three) of the survey asked participants, “what are your favourite foods?”, “what foods do you eat most often?”, and do you prefer eating local food (food grown or caught in the Solomon Islands) or shop foods (food that has been brought into the Solomon Islands from another country or processed, long life food), or both and why. Free listing is a method intended to generate data on the participant’s food preferences and commonly consumed foods [28]. Assuming that participants listed food items in order of familiarity and that the most commonly listed foods were consumed most often locally [28], this data was used to supplement the information collected on dietary diversity. Interviews were conducted in the participant’s language of choice, English, Pidjin or local dialect (or a combination). When participants responded in a language other than English, responses were translated into English by the local translator and recorded by the interviewer. Interviews took approximately 25 min to complete.

The tool was piloted in Auki with residents, reviewed and amended by the research team prior to commencement of data collection. Amendments were made to the language used in several questions to ensure they could be translated successfully by translators and interpreted appropriately by participants. A question pertaining to income was removed after two participants expressed unease in answering. Training was provided for researchers and translators to ensure coherent and consistent application of the tool. This included reviewing locally available foods, becoming familiar with different ingredients used in mixed dishes, discussing minimum quantities of foods and individual food items that could be classified into more than one food group. The tool was provided in English as all translators were comfortable interviewing in either language and translating into English for recording. Any answers that were not clear were discussed with the research team to clarify translation. Additionally, the local research team member assisted throughout the research design, training and data collection process, ensuring each step was culturally acceptable and information was interpreted appropriately. 

### 2.4. Data Analysis

Dietary diversity was determined by summation of the number of food groups recorded in the 24-h dietary recall against the 12 pre-determined food groups. A score of ‘1’ was assigned if a food from that group was consumed at least once, regardless of the quantity. A score of ‘0’ was assigned to any food group that was not consumed. Two researchers reviewed the categorisation of all foods to ensure consistency of coding. No issues with translation and coding of responses were identified due to the native speakers and Australian researchers working together to record responses. The individual dietary diversity score (DDS) for each adult was then calculated by summing the combined total food group scores.

The calculation of DDS according to the FAO’s guidelines was modified for this study to avoid excluding fats and oils, discretionary foods and spices, condiments and beverages. Calculation of DDS varies depending if the assessment is made at an individual or household level [27]. Individual assessment is dependent on nine food groups, and household assessment is dependent on 12. In this study, the individual was assessed, however, the analysis of 12 food groups is presented. When using the 9 food groups, oils and fats (group 10), discretionary foods (group 11) and spices, condiments and beverages (group 12) are excluded. However, these three food groups are important to consider in an individual diet due to their potential contribution of fat, sugar and salt. Consumption of these food substances contribute to the risk of developing non-communicable diseases [17,20], and according to previous dietary behavior data they are consumed frequently in the Solomon Islands [20], therefore the dietary diversity score is presented as a score out of 12. 

Statistical analyses were conducted using Statistical Package for Social Sciences (SPSS version 24, SPSS Inc., Chicago, IL, USA, 2016). Descriptive analysis including means, frequencies and percentages were used to measure socio-demographic characteristics, DDS and food preferences. Additionally, variables were created for individual food items listed in 24-h recalls and frequencies were measured to analyse the dietary diversity within food groups. The independent t test was used to determine whether there was a statistically significant difference between socio-demographic variables and dietary diversity scores.

Responses to the question “why do you prefer local/imported food” were analysed using a conventional content analysis approach whereby categories were coded directly from the text responses to this question [29].

To increase the trustworthiness and confirmability of the interpretation and analysis of qualitative data, one researcher coded all responses into themes and reviewed these with two other researchers. Any discrepancies were resolved through discussion with at least two researchers.

## 3. Results

### 3.1. Socio-Demographic Characteristics of Participants

A total of 133 participants from Auki town center (39.1%) and 12 surrounding villages (60.9%) with a mean age of 37.12 years (range: 18 to 72 years of age) took part in this study. More females (63.2%) than males (36.8%) participated. The majority of participants (90.2%) had completed some form of education, with (50%) reporting current employment (Table 1). Participants were from varying household sizes, with numbers fluctuating between 1 and 30 people over different periods of time. The current mean household size at the time of interview was 6 people. Several mixed forms of transport were used to access food from markets or stores, with people walking (n = 72), using a variety of public transport (bus, n = 44; truck, n = 8; taxi, n = 2), private canoe (n = 17) and private car (n = 7). Several of the characteristics of participants in this study are typical of Solomon Islands’ population data (average household size in Malaita = 5.6, women without education in the Solomon Islands = 9.2%, ratio of males = 36.6% and ratio of females = 63.4% in the Solomon Islands) [14,24].

### 3.2. Dietary Diversity Scores

Overall, participants’ DDS ranged between 2 and 12 food groups, with an average DDS of 7.27. Females had significantly higher dietary diversity scores (7.55 ± 1.609), compared to males (6.80 ± 1.803), t (131) = −2.486, *p* = 0.014. In addition, participants living in Auki town center had significantly lower dietary diversity scores (6.90 ± 1.706), compared to participants who lived in the surrounding villages (7.51 ± 1.689), t (131) = −1.999, *p* = 0.048. There were no statistically significant associations between DDS and all other socio-demographics; age, education, employment, access to garden, household size, main way of obtaining food, and if the participant ate food out (away from home).

### 3.3. Dietary Characteristics

A total of 96 individual food items were reported to be consumed. Most participants (>80%) reported consuming at least one food item from each of the cereals, vegetables, condiments, seafood and discretionary food groups (Table 2). Items from the tuber and roots, fruits, nuts, seeds and legumes, and fats and oils groups were consumed by between 35% and 70% of participants. By contrast, excluding seafood food items, only a small proportion (24.1%) of participants consumed foods of animal origin from the dairy, meat and eggs food groups. The variety of items consumed from each food group varied greatly, and in most instances only a few food items within each food group were consumed by more than 25% of participants. There were no food groups that all participants reported consuming at least one food from, however 94.7% ate some form of cereal, mostly white rice (88%). Other food items consumed by more than 50% of participants included tea (71.4%), cabbage (67.7%), sugar (60.2%), canned fish (57.1%) and salt (51.9%). 

Table 3 presents the percentage of people who consumed a food group from each DDS, indicating which food groups are added as DDS increases or decreases. As DDS increased, the proportion of participants who consumed foods from the tubers and roots, vegetables, fruit, eggs, nuts, seeds and legumes, seafood, condiments, and fats and oils food groups increased (Table 3). However, the proportion of participants who consumed cereal remained almost constant across each DDS (Table 3). 

### 3.4. Food Preferences

Participants listed a total of 44 unique “favourite foods” (n = 32 local foods), with each participant listing between one and eight food items. In response to the question “what do you eat most often?”, a total of 28 different food items were listed (n = 21 local foods), with each participant listing between one and four different food items. Any food items that were listed less than three times are not included in the table due to size considerations. Some participants listed “local food” and “garden food” as their favourite food rather than an individual food item. Therefore, the term “food items” in Table 4 includes individual foods and these particular food groups. In this instance the types of local and garden foods are not known.

The majority (91%) of participants indicated a preference for local food. Many participants listed multiple reasons for their preference (Table 5). In comparison, only 4.5% of participants reported a preference for shop food, and 4.5% preferred both shop and local food. Responses to why participants selected a preference for shop food included: “it’s nice”, “convenient”, “easy to prepare”, “tastes sweet” and “it’s easy”. Responses to why participants selected a preference for both shop and local food included: “provides a balance”, “depends on budget”, “because I have a choice”, “build my health”, “like a variety” and “both have nice food”.

## 4. Discussion

This study presents one of the first assessments of dietary diversity of an adult population of Solomon Islanders located on Malaita Island. Individual DDS for this population was shown to be 7.27, with a range of 2–12. While there is no consistent method to categorise DDS as high, medium or low in the Pacific environment, our results indicate that dietary diversity is limited in some of this population. Our results are consistent with findings shown in another Solomon Islands setting (Honiara), where mean individual DDS was 6.54 [10]. Although this is slightly lower than our cohort, Honiara produces only 10% of their food requirements, and are more reliant on cash purchases than Auki [7,10]. In comparison with other Pacific nations, DDS scores in the coral atoll nation of Kiribati are lower with only 3% of participants with a household DDS above seven and the majority (61%) with scores of four or below [19]. Lower dietary diversity in Kiribati was associated with a reliance on refined rice and flour products, and was linked with multiple micro-nutrient deficiencies [19]. Despite the agricultural variances between these countries, there is a demonstrated link between lower DDS and reliance on commercial food products in the Pacific region, which may potentially be associated with micronutrient deficiencies and trends in malnutrition. 

In this study, women were more likely to have a higher DDS compared to males. There is limited literature on the Pacific regarding the relationship between DDS and gender, however, research undertaken in various low and middle-income countries (Nepal, Cambodia and Ghana) has reported that a woman’s domestic work and amount of time spent cooking was positively associated with higher dietary diversity [30]. In contrast, working long hours in agriculture-based activities in Mozambique was negatively associated with women’s dietary diversity [30]. Women in the Solomon Islands play a central role in the domestic household and local food systems, and are generally responsible for growing, cultivating, selling, purchasing, processing and preparing food for consumption [7,31]. In this study more men reported employment, but more women reported access to food from a garden. These results are potentially connected to gender roles (such as unpaid agricultural labour) [7] that provide women with wider exposure to a variety of foods, which increases opportunities for consumption [30,31]. Examining the link between women’s domestic and agricultural duties was outside the scope of this study, however, further investigation into potential associations between work and DDS in the Solomon Islands is warranted. Understanding gender-based differences could direct initiatives to improve nutrition while considering the social and economic differences between genders.

We found that individuals who lived further from the Auki town center were more likely to have a higher DDS. According to the FAO’s Guidelines for Measuring Household and Individual Dietary Diversity, dietary diversity is often much greater in urban centers compared to rural areas, where there is better access to adequately supplied food markets [27]. While Auki has a central market and other retail/food service venues to purchase food (i.e., small convenience stores), food prices have been rising in the Solomon Islands since the global financial crisis in 2007, reportedly having more impact on the urban populations ability to purchase foods [7]. Given the focus on local food systems on the island of Malaita, it is plausible that those individuals living further from the town center also have increased access to more food items through gardening, hunting and fishing at a lower cost or through trading with others, potentially increasing DDS [10]. 

The results from our study indicate that consumption of the food groups; nuts, seeds and legumes, meat, egg, and milk and other dairy products were low in this population. Items from the cereals, condiments, vegetables, seafood, and discretionary food groups were consumed the most. Apart from the vegetables food group, our findings are similar to those reported for the Solomon Islands [10] and other Pacific settings [19,32] and reflect the food environment in Auki (i.e., limited access to meat and dairy products (unpublished results). Our findings also suggest that a nutrition transition is underway in this region [7]. 

While DDS is based on food group consumption, the variety of foods consumed from within each food group is also important to consider [33]. A diverse diet could contain a range of low quality foods high in fat, salt and processed sugars or a range of nutrient dense foods, making it vital to determine which food groups are lacking in quality foods [33]. Our results demonstrate that consumption from each food group was dominated by a limited number of food items. The condiments group had a higher variety of food items, however, most of these contained added salt, which can be harmful to health when overconsumed [17]. This study found there was a lack of variety in wholegrain cereals, fruits and vegetables, all important contributors of fibre and micro nutrients to the diet [17,34]. A lack in diversity of these food items may increase the risk of multiple micro nutrient deficiencies and dietary related diseases [17,34], while high intakes of sodium (found in many of the condiment items) and low intakes of whole grains are two of the leading dietary risk factors for mortality and disability affected life years (DALYS) worldwide [17].

Fish was the most frequently reported animal food source, predominantly canned, followed by fresh fish. All other animal food sources were consumed by less than 20% of participants. Another study based in Tuvalu, found that participants preferred fresh fish, but consumed more canned fish [28], which appears to be a trend across various countries in the Pacific region [9,10,19,35]. In most Pacific Island countries, fish consumption is a major source of animal protein and fishing contributes to food security through income generation [9,21,22,35]. However due to a rapidly increasing population, environmental issues, pollution and insufficient coastal resources, fish prices are increasing, and consumption is decreasing; this influences the ongoing nutrition and food security problems in the Solomon Islands [9,22,35]. In contrast, canned fish is cheaper, easier to use, less perishable (compared to fresh product) and readily available [9,10], which may explain its higher consumption by this group. However, higher amounts of added sodium and oil in canned fish compared to fresh fish can pose added public health risk, as such, promotion of brined varieties of canned fish is warranted to reduce fat and energy content. Our results suggest that participant’s diets are not only lacking diversity due to a lower variety of foods reportedly consumed from each food group, but also, they may lack quality.

Our results suggest that a more diverse diet, consisting of predominantly locally grown fresh food is more desirable amongst study participants. Moreover, a majority of participants (91%) indicated a preference for local food over shop food, with the majority (71.9%) noting desirable health benefits from the consumption of local foods. However, in reality participants reported diets that consisted of only a few different food items, and out of the 15 food items consumed by more than 25% of participants, just over half of these (n = 8) were processed shop foods (white rice, white bread, sugar, canned fish, coconut milk, coconut cream, tea and salt). A recent study in Honiara found that while participants understood a traditional diet was healthier, these foods were considered unaffordable and less convenient [10]. Additionally, Fijians have been reported to remark if they did not grow their own vegetables then they could not afford to buy them [36]. Given the high level of unemployment observed in our study population and apparent over-reliance on commercial food sources in Solomon Island urban populations [11], it is likely that our participants make dietary choices driven by financial motives; opting to purchase foods such as white rice which will last longer and feed a greater number of people. Comparatively, a study in Malaita observing the relationship between food security and the logging industry found that when participants gained money from logging employment, they were less likely to eat fresh produce and more likely to spend their money on store food [22]. This store food was often perceived to be popular due to its practical value, with some women reporting that it made life easier by saving time and energy when cooking meals [22]. Poor cohorts with access to a home garden will likely consume local foods, but if they have no access to a home garden, they may opt for the cheapest food available and when financial resources are highest, they opt for convenience. Further research on the food choice behaviours of this population is warranted. 

Participants in this study voiced a desire for greater food variety than what they were consuming. In response to “what foods do you eat most often”, the most frequently listed foods were similar to the most frequently reported foods in the dietary diversity questionnaire. However, this trend was not reflected in response to “what are your favourite foods”, rather, participants listed a greater variety of alternative foods. A similar free listing study with adults in Tuvalu (a Pacific Island nation) found comparable results, stating that people are more likely to list items in order of familiarity, which reflects a general preference and consumption of those foods [28]. 

Given the small comparative survey sampling, findings presented in this paper should be considered as a preliminary assessment of the dietary behavior in Auki. We recruited participants in, and near the main town center which also limits the generalisability of our results to those living in other areas of Malaita and the Solomon Islands. The DDS measure relies on a 24-h recall process. This process has recognised limitations because it relies on participant recall and one 24-h recall period that does not necessarily reflect habitual dietary intake or consider diversity in food availability and consumption during different seasons. Additionally, the results cannot be used to reflect nutrient adequacy because the quantity of foods eaten was not included in the calculation of DDS. There is a small risk that responses to later questions were influenced by the time taken to complete the questionnaire. Despite these limitations with the tool used, other tools for assessing dietary intake in the Pacific region are limited. Development and validation of a more appropriate assessment tool specific to the diets, food system and cultural norms in the Pacific region is necessary. Given that there are a few prior published assessments of Malaita and no prior studies in Auki this study makes an important contribution to the literature. 

## 5. Conclusions

Dietary diversity in this study population was limited, with notable differences in DDS based on gender and population location. Given the preliminary nature of the current study, further work is required to better understand the drivers behind gender-based dietary behavior in Auki, and further explore spatial differences in DDS based on the inclusion of remote communities. Taking a transdisciplinary approach to work across the interconnected elements of the food system in the Solomon Islands is needed to better understand how political, physical and social environments influence dietary intake. Political interventions that foster the trade of produce between remote islands may increase the availability of a variety of foods that will increase dietary diversity, as well as promote broader social and economic benefits across the food system. Further research and development efforts that take a systems approach are warranted to consider the contribution of gender, geographical location, food access and availability in influencing dietary diversity in the Solomon Islands. 

## Figures and Tables

**Table 1:** Participants’ sociodemographic characteristics (n = 133).

Characteristic	Female	Male	Total
*Age*			
*Mean*	35.6747	39.6250	37.12
*Standard Deviation*	12.2	13.0	12.6
18–24	18.1%	14.3%	16.7%
25–34	33.7%	22.4%	29.5%
35–50	34.9%	46.9%	39.4%
51–70	13.3%	14.3%	13.6%
70+	-	2.0%	0.8%
*Residence*			
Auki town center	36.9%	42.9%	39.1%
Surrounding villages	63.1%	57.1%	60.9%
*Education level*			
No education	10.7%	8.2%	9.8%
Primary	35.7%	28.6%	33.1%
Secondary (form 1–3)	17.9%	20.4%	18.8%
Secondary (form 4–6)	25%	20.4%	23.3%
Secondary (form 7)	1.2%	4.1%	2.3%
Technical institute	1.2%	6.1%	3.0%
University	8.3%	12.2%	9.8%
*Occupation*			
Unemployed	55.4%	40.8%	50%
Employed	44.6%	59.2%	50%
Access to garden *			
Yes	67.1%	63.3%	65.6%
No	32.9%	36.7%	34.4%
*Main way of obtaining food in past 24 h*			
Self-provided	26.2%	32.7%	28.6%
Store/road side vendor	32.1%	30.6%	31.6%
Exchanged or gifted	1.2%	-	0.8%
Market	40.5%	36.7%	39.1%
*Ate food out (away from home) in previous 24 h*			
Yes	34.5%	26.5%	31.6%
No	65.5%	73.5%	68.4%

* Additionally, 67.9% (n = 55) of participants who lived in surrounding villages had access to a food garden, compared to 60% (n = 30) of those who live in Auki town center.

**Table 2:** Variety of all food items consumed from each food group and proportion of food group consumption by males and females.

Food Group	Females	Males	Total Proportion	Food Items
Cereals	95.2%	95.9%	94.7%	White Rice ^2^, white bread ^2^, white flour, noodles, savoury cracker, weetbix, popcorn
Condiments	89.3%	89.8%	89.5%	Tea ^2^, salt ^2^, coffee, alcohol, pepper, soy sauce, curry seasoning, umami seasoning, garlic, ginger, chilli, chicken flavouring, noodle flavouring, oyster sauce, mushroom sauce, tomato sauce, chilli sauce
Vegetables	90.5%	83.7%	88%	Sweet potato ^2^, tomato ^2^, cabbage ^2^, cucumber ^2^, pumpkin, watercress, taro leaf, cassava leaf, pumpkin tips, lettuce, snake beans, eggplant, capsicum, mangrove root, spring onion, onion
Seafood ^1^	86.9%	79.6%	84.2%	Canned fish ^2^, fresh fish ^2^, crab
Discretionary	82.1%	83.7%	82.7%	Sugar ^2^, cake, candy, donut, sweet bun, sweet biscuit, chips, ice block, soft drink, sweet drink, milo
Fats & Oils	77.4%	53.1%	68.4%	Coconut milk ^2^, coconut cream ^2^, coconut oil, palm oil, peanut oil, vegetable oil, butter, other cooking oil
Fruit	71.4%	63.3%	68.4%	Mandarin, pawpaw, mango, watermelon, starfruit, pomelo, banana, lemon, lime, pineapple, coconut, potera, guava, soursop, avocado, local apple and cherry
Tubers & Roots	67.9%	59.2%	64.7%	White potato ^2^, yam, taro, cassava, breadfruit, plantain
Nuts, Seeds & Legumes	46.4%	28.6%	39.8%	Ngali nuts ^2^, peanuts, peanut butter, kat nuts
Dairy ^1^	17.9%	20.4%	18.8%	Milk powder, ice cream
Meat ^1^	15.5%	12.2%	14.3%	Sausage, beef steak, pork, chicken
Eggs ^1^	15.5%	10.2%	13.5%	Poultry Eggs

N = 133. ^1^ Foods of animal origin. ^2^ Foods that were consumed by >25% of participants.

**Table 3:** Proportion of consumption from different food groups by dietary diversity score.

DDS	1–3	4	5	6	7	8	9	10	11	12
No of adults	2	5	11	26	26	33	21	4	4	1
% of adults	1.6%	3.8%	8.3%	19.5%	19.5%	24.8%	15.8%	3%	3%	0.8%
Cereals	50%	100%	90.9%	96.2%	96.2%	97%	95.2%	100%	100%	100%
Tubers/Roots	50%	20%	27.3%	42.3%	61.5%	81.8%	90.5%	75%	100%	100%
Vegetables	100%	40%	45.5%	88.5%	88.5%	97%	100%	100%	100%	100%
Fruit	-	20%	45.5%	46.2%	65.4%	81.8%	95.2%	100%	100%	100%
Meat	-	-	-	15.4%	7.7%	24.2%	14.3%	-	25%	100%
Eggs	-	-	-	-	3.8%	15.2%	23.8%	75%	75%	100%
Seafood	50%	60%	81.8%	76.9%	88.5%	87.9%	85.7%	100%	100%	100%
Nuts	-	-	9.1%	15.4%	26.9%	48.5%	76.2%	100%	100%	100%
Milk/dairy	-	-	9.1%	7.7%	26.9%	6.1%	28.6%	50%	100%	100%
Fats/Oils	-	20%	45.5%	46.2%	73.1%	75.8%	95.2%	100%	100%	100%
Discretionary	-	80%	54.5%	76.9%	73.1%	93.9%	100%	100%	100%	100%
Condiments	-	80%	90.9%	88.5%	88.5%	90.9%	95.2%	100%	100%	100%

**Table 4:** Free listing results for “favourite food”, preference and reported frequency of “most often consumed food items” listed by three or more participants.

Food Groups	Food Items (Listed by Three or More Participants)	Frequency
*What are your favourite foods?*		
Vegetables	Sweet potato	34.6%
	Cabbage	18.8%
	Taro leaf	5.3%
	Pumpkin	3%
	Cucumber	3%
	Mangrove root	2.3%
Seafood	Fresh fish	30.8%
	Canned fish	2.3%
Roots and tubers	Taro	12.8%
	Cassava	9%
Cereals	Rice	9.8%
Meat	Chicken	6%
Local Food	Local food	3.8%
Fruit	Watermelon	3%
	Banana	3%
*What do you eat most often?*		
Cereals	Rice	63.9%
Roots and tubers	Potato *	28.6%
	Taro	3.8%
	Cassava	3%
Vegetables	Cabbage	18.8%
	Pumpkin	3%
Seafood	Canned fish	8.3%
	Fresh fish	5.3%
Garden Food	Garden food	3%

* Classification of which potato was not provided by all participants, therefore “potato” includes white potato and sweet potato.

**Table 5:** Conventional content analysis for local food preference, including common themes and two examples of why from each theme.

Theme	Frequency	Examples
Health	71.9%	“It’s healthy”
		“Makes body strong”
Natural (from the garden)	16.5%	“It’s free from my garden”
		“Comes from the ground”
Taste, freshness and variety	14.9%	“Better taste then rice”
		“I eat mostly rice and want to eat fresh, local food to change it up”
Affordability	12.4%	“Can’t afford shop food”
		“Less expensive”
Availability and convenience	5.8%	“Easy to get and easy to cook”
		“Local food is best and easy to find”
Tradition	4.1%	“We grow up with it”
		“Traditional food”
Mistrust of shop food	2.9%	“Food from the shop makes us sick”
		“Shop food has too many unknown ingredients”
